# Understanding the Public Discussion About the Centers for Disease Control and Prevention During the COVID-19 Pandemic Using Twitter Data: Text Mining Analysis Study

**DOI:** 10.2196/25108

**Published:** 2021-02-09

**Authors:** Joanne Chen Lyu, Garving K Luli

**Affiliations:** 1 Center for Tobacco Control Research and Education University of California, San Francisco San Francisco, CA United States; 2 Department of Mathematics University of California, Davis Davis, CA United States

**Keywords:** COVID-19, CDC, Twitter, public discussion, public opinion, social media, data mining

## Abstract

**Background:**

The Centers for Disease Control and Prevention (CDC) is a national public health protection agency in the United States. With the escalating impact of the COVID-19 pandemic on society in the United States and around the world, the CDC has become one of the focal points of public discussion.

**Objective:**

This study aims to identify the topics and their overarching themes emerging from the public COVID-19-related discussion about the CDC on Twitter and to further provide insight into public's concerns, focus of attention, perception of the CDC's current performance, and expectations from the CDC.

**Methods:**

Tweets were downloaded from a large-scale COVID-19 Twitter chatter data set from March 11, 2020, when the World Health Organization declared COVID-19 a pandemic, to August 14, 2020. We used R (The R Foundation) to clean the tweets and retain tweets that contained any of five specific keywords—cdc, CDC, centers for disease control and prevention, CDCgov, and cdcgov—while eliminating all 91 tweets posted by the CDC itself. The final data set included in the analysis consisted of 290,764 unique tweets from 152,314 different users. We used R to perform the latent Dirichlet allocation algorithm for topic modeling.

**Results:**

The Twitter data generated 16 topics that the public linked to the CDC when they talked about COVID-19. Among the topics, the most discussed was COVID-19 death counts, accounting for 12.16% (n=35,347) of the total 290,764 tweets in the analysis, followed by general opinions about the credibility of the CDC and other authorities and the CDC's COVID-19 guidelines, with over 20,000 tweets for each. The 16 topics fell into four overarching themes: knowing the virus and the situation, policy and government actions, response guidelines, and general opinion about credibility.

**Conclusions:**

Social media platforms, such as Twitter, provide valuable databases for public opinion. In a protracted pandemic, such as COVID-19, quickly and efficiently identifying the topics within the public discussion on Twitter would help public health agencies improve the next-round communication with the public.

## Introduction

Since first identified in Wuhan, China, in December 2019, COVID-19 has spread rapidly around the world. On March 11, 2020, the World Health Organization (WHO) declared the coronavirus outbreak a pandemic and was unable to determine the duration of the pandemic [1]. As of August 5, 2020, 213 countries and territories around the world have reported a total of 18,939,540 confirmed cases and a death toll of 709,700 [2]. The United States reported its first case on January 20, 2020 [3]; the country had 4,802,491 total COVID-19 cases and 157,631 total deaths as of August 5, 2020 [4].

The Centers for Disease Control and Prevention (CDC) is the “nation’s health protection agency, working 24/7 to protect America from health and safety threats” [5]. Since January 21, 2020, the CDC has launched an agencywide response to this pandemic, including preparing health care providers and health systems, supporting governments at various levels on the front lines, and learning and sharing COVID-19 knowledge via a variety of communication channels. Amid the unprecedented public health crisis caused by the pandemic, the bewildered public in dire need of guidance depends on the quick response of public health authorities and is in greater demand for information and advice from them [6]. Previous studies found that the public willingness to take the advice (eg, handwashing) proposed by public health agencies, which will further impact the success of disease control strategies and policies, is related to the trust that the public has in the agencies [7-12]. During the Zika outbreak, studies found a substantial topic discrepancy between public concern and the CDC’s response to Zika [13-16], undermining the efficacy of the CDC’s Zika control efforts. Considering that the COVID-19 pandemic will last for a protracted time, timely information about public opinion regarding the CDC’s COVID-19 response efforts and their concerns about COVID-19 can provide insight to improve the next round of communication with the public.

Social media platforms such as Twitter have not only become increasingly important for the public to seek, share, and discuss information, but have also provided valuable platforms for the surveillance of public opinion, allowing for the monitoring of the public’s questions, concerns, and responses to health threats [17-19]. In previous public crises, such as the Ebola virus [20], Zika virus [13,17,21], and H1N1 virus outbreaks [22], Twitter was used as an up-to-date information source to gauge the public’s knowledge and reactions to the epidemics. During the Zika outbreak, it was found that inaccurate information proliferated on social media, and conspiracy theories regarding the Zika virus were more popular than public health education materials from health agencies [23]. Therefore, using social media to monitor public knowledge, to evaluate the information spread online, and to address the identified problems in a timely manner is crucial in battling public health crises. In addition, when an epidemic situation is not clear, the discussion on social media can provide timely information for improving epidemic surveillance and forecasting [24-27]. Therefore, the value of online discussion on social media platforms, especially during infectious disease epidemics, has gained ever more attention by public health agencies and officials [24-26]. The CDC has been actively using Twitter to reach out with timely health and safety information [28] and even hosted live Twitter chats to directly communicate with the general public in the periods of the Ebola outbreak [20] and the Zika outbreak [13].

The ongoing COVID-19 pandemic demands continuous and evolving efforts of using social media data to understand the public’s thoughts and concerns. While a series of studies made significant contributions along these lines, the majority of existing studies analyzed data collected before the middle of April 2020 [29-39]. In addition, there have been scarce social media studies with a focus on the CDC during the COVID-19 pandemic [6], the main source for evidence-based information about the pandemic [40]. To fill the gaps in knowledge after the WHO’s declaration of a pandemic, we used text mining methods to analyze COVID-19-related tweets about the CDC from March 11 to August 14, 2020. By doing so, this study identified the topics emerging from the tweets and the overarching themes of these topics, shedding light on a series of questions: What are the public concerns over COVID-19? What does the public expect from the CDC? and How does the public comment on the current performance of the CDC in response to COVID-19?

## Methods

### Data Extraction and Preprocessing

The IDs from a total of 128,432,021 tweets, without retweets, from March 11 through August 14, 2020, were obtained using the data set maintained by Georgia State University’s Panacea Lab [41]. These tweets were collected by the Panacea Lab using the following 13 keywords: COVD19, CoronavirusPandemic, COVID-19, 2019nCoV, CoronaOutbreak, coronavirus , WuhanVirus, covid19, coronaviruspandemic, covid-19, 2019ncov, coronaoutbreak, and wuhanvirus. Since Twitter's Terms of Service do not allow the full JavaScript Object Notation (JSON) for data sets of tweets to be distributed to third parties, Georgia State University's Panacea Lab can only provide tweet IDs [42], which can be hydrated to obtain the JSON objects from these tweets.

During the tokenizing stage, we used the *gsub* function in R (The R Foundation) to extract the tweets whose language field in the tweets’ metadata was specified as English. All text mining was done using R 4.0.2 GUI (graphical user interface) 1.72 Catalina build (7847) on a Mac running 10.15 Catalina.

**Figure 1:**
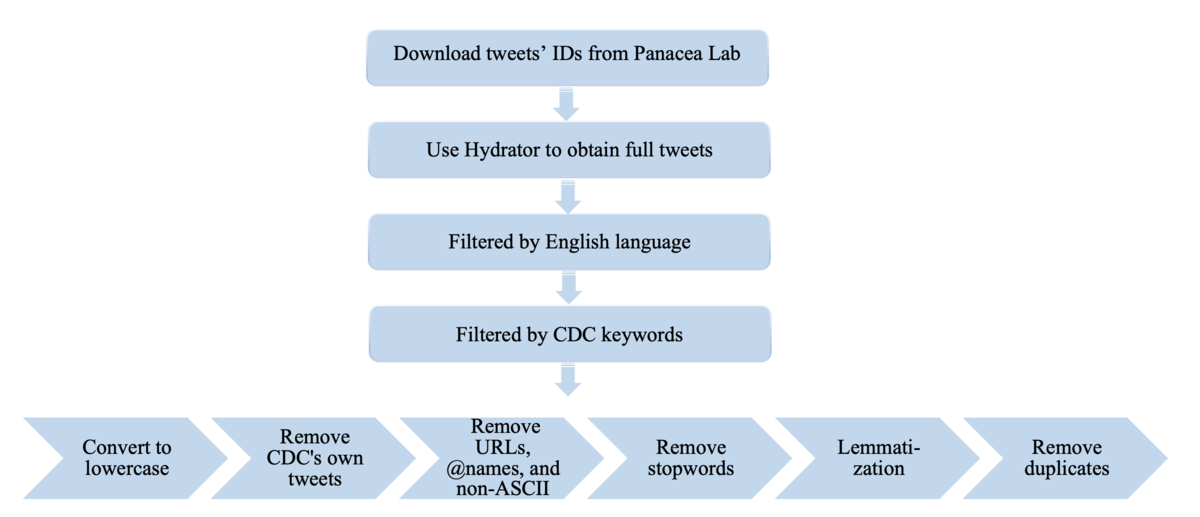
Flowchart for data extraction and preprocessing. CDC: Centers for Disease Control and Prevention.

We converted all the tweets to lowercase and created a script to remove the URLs, mentioned names, non-ASCII (American Standard Code for Information Interchange) characters such as emojis, and anything other than English letters or spaces (eg, “1,” “?,” etc). Using the R package *dplyr*, version 1.0.2, we cleaned the tweets by removing duplicates, retained only tweets that contained any of five specific keywords—cdc, CDC, centers for disease control and prevention, CDCgov, and cdcgov—and eliminated all 91 tweets posted by the CDC itself. The final cleaned data set consisted of 290,764 unique tweets from 152,314 different users. We further cleaned the tweets by removing words and characters that were of little or no analytical value (eg, “the,” “very,” “&,” etc). We performed this task by creating our own list of stop words by appending the 13 keywords related to “COVID19” and the five keywords related to “CDC” to the English stop words list from the R package *tidytext*, version 0.2.6; this was done because we already knew that every tweet would contain one or more of those keywords, and having them in the tweets does not contribute to further our understanding of the main content of the tweets. Lastly, we stemmed and lemmatized the words to their root forms using the R package *textstem*, version 0.1.4 (eg, *studying*, *studies*, and *studied* were converted to *study*). See Figure 1 for a summary of our data extraction and preprocessing procedure.

### Topic Modeling

Topic modeling provides an automatic, or unsupervised, way of summarizing a large collection of documents. It can help discover hidden themes in the collection, group documents into the discovered themes, and summarize the documents by topic. Topic modeling is often referred to as *soft* clustering, but it is more robust and provides better and more realistic results than typical clustering (eg, k-means clustering) or *hard* clustering [43]. A typical clustering algorithm assumes a distance measure between topics and assigns one topic to each document, whereas topic modeling assigns a document to a collection of topics with different weights or probabilities without any assumption on the distance measure between topics. There are many topic models available. “The most widely used model for topic modeling is the latent Dirichlet allocation (LDA) model” [43], developed by David Blei, Andrew Ng, and Michael I Jordan in 2002 [44].

**Figure 2:**
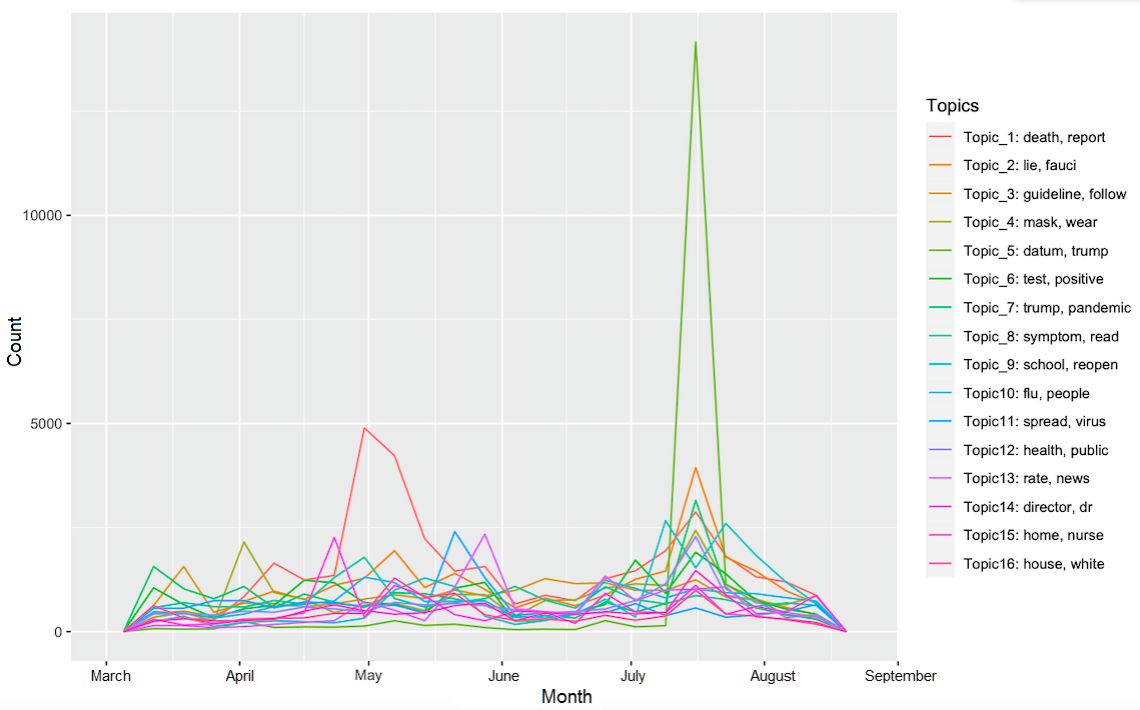
Weekly frequency of each topic on Twitter, from March 11 to August 14, 2020.

To extract common topics from this sheer number of tweets, we used the LDA algorithm for topic modeling. We performed the LDA algorithm on the data using the R *textmineR* package, version 3.0.4. The LDA algorithm requires manually inputting the number of expected topics. We ran the LDA algorithm on the data by varying the topic number from 2 through 40. For each topic number, we calculated the coherence score using the *textmineR* package; we ended up choosing 16 topics for the final model, as the topic number that was equal to 16 yielded the highest coherence score. The top eight terms from each of the 16 topics were generated. We also used the *geo_freqpoly* function in the R package *ggplot2*, version 3.3.2, to generate the frequency polygons (see Figure 2) in order to visualize the weekly frequency of the 16 topics from March 11 to August 14, 2020. For each tweet, the LDA assigned a probability to each of the 16 topics. We assigned the topic with the highest probability to a tweet and we grouped the tweets according to the most prevalent topics. To obtain representative tweets for each topic, we randomly sampled 100 tweets from each topic; the two authors then independently examined the sampled tweets, followed by a group discussion to select the most representative ones. If one of the authors thought that there were no conspicuous topics that emerged from the first 100 sampled tweets, another 100 tweets would be sampled and further reviewed; the authors continued this process until the two judged that there was a clear common topic and they reached a consensus. We used the *textmineR* package’s topic label function to generate an initial labeling for the topics. After carefully reading through the sampled tweets from each topic, the two authors refined the machine-generated labeling to give each topic the most accurate, concise, and coherent description (see Table 1). Through discussions, the authors further grouped the topics into overarching themes.

## Results

### Overview

The Twitter data generated 16 topics that the public linked to the CDC when they talked about COVID-19. Table 1 shows the topics generated, the number and percentage of each topic, description of topics, and the representative tweets. The topics are sorted according to the number of the tweets in decreasing order. Among the topics, the most discussed was COVID-19 death counts, accounting for 12.16% of the total tweets included in the analysis, followed by general opinions about the credibility of the CDC and other authorities and the CDC’s COVID-19 guidelines, with over 20,000 tweets for each topic. The topics in Table 1 can be categorized into four overarching themes, as discussed in the four sections following the table.

**Table 1:** COVID-19-related tweets about the Centers for Disease Control and Prevention (CDC) by topic.

Topic No.	Terms contributing to topic model	Total tweets (N=290,764), n (%)	Description	Examples of representative tweets (date posted)
1	Death, report, count, week, numb, total, datum, and pneumonia	35,347 (12.16)	Discussion of COVID-19 death counting, focusing on whether the accounting of COVID-19 cases is accurate	“Actually, they aren’t even lying about it. The CDC was frank about changing their accounting of COVID-19 cases to encompass contacts with presumptive cases, statistically causing this sudden huge case explosion. [link]” (July 13, 2020) “COVID death counts are inflated. According to CDC: ‘ideally testing for COVID-19 should be conducted but it is acceptable to report COVID-19 on a death certificate without this confirmation if the circumstances are compelling within a reasonable degree of certainty.’ Read and rt! [link]” (August 7, 2020)
2	Lie, Fauci, people, Trump, trust, doctor, listen, and vaccine	26,026 (8.95)	General opinions about credibility of the CDC and other authorities	“Coronavirus is not political. the curve has not flattened and people are still dying. Trump and our leaders need to listen to scientists and the CDC. You are, most definitely, a cultist.” (July 10, 2020)(July 28, 2020) “People: I don't believe anything about the coronavirus I hear from the government, Dr. Fauci, CDC, FDA, WHO, JLA, OPP, or anyone else I've ever heard of. Nope.” (July 28, 2020)”
3	Guideline, follow, follow_guideline, people, stay, recommendation, social, and distance	20,032 (6.89)	CDC’s COVID-19 guidelines, with a considerable number of tweets about the social distancing and stay-at-home orders	“Bottom line: the more adherent we are to the CDC guidelines, the faster the economy will recover. Stop gathering, put masks on and we will get out of this. Labor secretary: discipline now in following COVID-19 guidelines will end economic slump 'quickly'. [link]” (April 4, 2020) “Coming back to work after 3 months stay-home order (COVID-19) following all safety measures CDC guidelines. Safety and health of staff and clients are utmost importance. #COVID19 #safetyfirst #von #mianailspa. [link]” (June 24, 2020)
4	Mask, wear, wear_mask, spread, recommend, people, cloth, and public	19,934 (6.86)	CDC’s recommendation of wearing face masks to slow the spread of virus	“@[tag] @[tag] @[tag] well, here's the us’ CDC giving guidelines on how to convert a bandana or even a t-shirt to a face covering. gl! [link]“ (April 11, 2020) “CDC recommends people wear cloth face coverings in public settings when around people outside of their household, especially when other social distancing measures are difficult to maintain. [link]” (June 29, 2020)
5	Datum, Trump, hospital, administration send, report, Trump_administration, and control	19,098 (6.57)	Commenting on COVID-19 data reporting being routed away from the CDC	“@[tag] let's make sure coronavirus reporting isn't politicized. If data isn't going to the CDC it needs to be transparent. What a crazy move in the middle of a pandemic!” (July 15, 2020) “This is a very bad thing: white house strips CDC of data collection role for COVID-19 hospitalizations. [link]” (July 15, 2020)
6	Test, positive, antibody, test_positive, result, virus, kit, and antibody_test	18,937 (6.51)	Tweets focusing on the accuracy and inaccuracy of antibody tests	“@[tag] @[tag] CDC? the agency that used COVID19 tainted tests in the beginning of February? Where did CDC get the tests?” (April 22, 2020) “@[tag] @[tag] @[tag] haha from the CDC.... the CDC also say that a positive antibody test may not be ‘COVID19’ and may be an antibody picked up from a virus such as the common cold.... most of these tests are faulty....” (July 4, 2020)
7	Trump, pandemic, response, fund, test, cut, president, and administration	18,888 (6.50)	Commenting on the Trump administration’s health care policies, focusing on cutting CDC funding and dismantling the pandemic response team	“@[tag] @[tag] President Obama created the best pandemic unit in the world. The whole world looked at it as shining example. That pandemic teams' purpose was to secure preparedness for the USA! Republicans fired them in 2018, cut funds for CDC and gave tax breaks to rich!” (March 16, 2020) “The Trump administration is trying to block funding for coronavirus testing and contact tracing, as well as for the CDC, in the upcoming coronavirus relief bill [link]” (July 18, 2020)
8	Symptom, read, update, pandemic, virus, outbreak, guidance, and list	18,326 (6.30)	CDC adding new symptoms of COVID-19 to its list: six symptoms in April and three in June 2020	“The us centers for disease control and prevention has added six new symptoms of COVID-19 to its list: chills, repeated shaking, muscle pain, headache, sore throat, new loss of taste or smell.” (April 28, 2020) “New post!!! Follow the link provided World_News CDC: here are 3 ‘new’ COVID-19 coronavirus symptoms to make 12- Forbes [link]” (June 27, 2020)
9	School, reopen, child, risk, guideline, kid, guidance, and report	18,201 (6.26)	Worries about reopening schools, and discussion of children’s risk of COVID-19	“Exactly why we shouldn't be opening schools before they can meet the CDC requirements. [link]” (July 18, 2020) “And this is what returning to school will look like...260 at Georgia overnight camp test positive for coronavirus, CDC says [link]” (August 2, 2020)
10	Flu, people, die, death, million, American, vaccine, and month	16,950 (5.83)	Comparing the number of COVID-19 deaths with the number of flu deaths	“COVID-19 has led to more than 454,000 illnesses and more than 20,550 deaths worldwide. In the US alone, the flu (also called influenza) has caused an estimated 38 million illnesses, 390,000 hospitalizations and 23,000 deaths this season, according to the CDC.” (April 11, 2020) “@[tag] according to the CDC there were between 12k and 61k flu deaths per year over the past 10 years. Taking the worst year (which was an outlier) COVID-19 has killed roughly 3x as many victims in half the time. COVID-19 is not the flu!!” (August 10, 2020)
11	Spread, virus, China, surface, travel, easily, Wuhan, and January	15,223 (5.24)	A wide range of discussion surrounding the spread of COVID-19: China’s warning of human-to-human transmission in January 2020, travel restrictions, and surface spread	“Coronavirus ‘does not spread easily’ by touching surfaces or objects, CDC says. But it still ‘may be possible.’ [link] via @[tag] possible but not likely - time to get over it. like any germ.” (May 21, 2020) “Once people start traveling again, the risk of transmission will surge. ‘It keeps me up at night,’ CDC's Dr. Cochi in @[tag] about growing immunity gaps for measles, polio and other vaccine-preventable diseases as countries pause vaccination campaigns to mitigate #COVID19 spread [link]” (June 16, 2020)
12	Health, public, datum, public_health, government, report, and agency	15,010 (5.16)	Tweets discussing the CDC not leading the control over COVID-19	“With the white house now having all COVID-19 data and not allowing the CDC to monitor it, the information will no longer be provided to the public. This is a scary time where the government will no longer tell us about the pandemic.” (July 16, 2020) “US government health advisers say hospitals are ‘scrambling’ after Trump administration's ‘abrupt’ change to COVID-19 data reporting requirements – ‘it’s another example of CDC being sidelined’. @[tag], told @[tag] [link]” (August 14, 2020)
13	Rate, news, death_rate, death, estimate, low, infection, and report	13,963 (4.80)	Death rate of COVID-19, with a considerable number of tweets emphasizing the low death rate	“Best estimate is 0.4 percent death rate for COVID-19 patients with symptoms: CDC [link]” (May 22, 2020) “The CDC just confirmed that #COVID19 has a 0.2% fatality rate, which is lower than the seasonal flu. Some other news you may have missed while being intentionally distracted [link]” (June 11, 2020)
14	Director, Dr, Redfield, warn, Fauci, wave, bad, and Robert	13,393 (4.61)	Quoting CDC director Dr Robert Redfield’s statements, especially the warning of a deadlier second wave	“CDC director warns that COVID-19 could return in winter combined with flu in deadlier second wave - Q13 Fox News [link]” (April 22, 2020) “CDC director: ‘The fall and the winter of 2020 and 2021 are going to be the probably one of the most difficult times that we experienced in American public health.’[link]” (July 15, 2020)
15	Home, nurse, patient, health, care, nurse_home, hospital, and official	10,906 (3.75)	Comments on the practice of sending COVID-19 patients to nursing homes	“#reopennj #openamericanow #trump2020 Murphy administration ignored advice and sent COVID-19 patients to nursing homes | mulshine [link]” (May 22, 2020) “5 governors ordered nursing homes to take COVID-19 patients that caused thousands of deaths, for which they now blame CDC and Trump: CA gov. Gavin Newsom. NY gov. Andrew Cuomo. NJ gov. Phil Murphy. MI gov. Gretchen Whitmer. PA gov. Tom Wolf.” (June 23, 2020)
16	House, white, White_House, force, task, task_force, Trump, and CNN	10,530 (3.62)	Tension between the White House and the CDC, focusing on CDC being sidelined in COVID-19 fight	“The White House’s coronavirus task force response coordinator, Deborah Birx, said in a recent meeting that ‘there is nothing from the CDC that I can trust,’ the Washington Post reported. Surprised?” (May 12, 2020) “#trumpviruscoverup #trumpfailedamerica #trumpisalaughingstock Dr. Rich Besser: CDC ‘sidelined’ from role as leader in #COVID19 fight [link]” (July 18, 2020)

### Theme 1: Knowing the Virus and the Situation

Based on the number of tweets, the most tweeted theme was knowing the virus and situation (132,139/290,764, 45.45%). This theme consisted of seven topics: discussion of COVID-19 death counting (35,347/290,764, 12.16%), accuracy of antibody test (18,937/290,764, 6.51%), new symptoms added to the list of COVID-19 (18,326/290,764, 6.30%), number of COVID-19 deaths (16,950/290,764, 5.83%), spread of COVID-19 (15,223/290,764, 5.24%), death rate of COVID-19 (13,963/290,764, 4.80%), and the CDC director Dr Robert Redfield's warning of a deadlier second wave (13,393/290,764, 4.61%). This theme reflected the public's desire to know the virus, such as how it spreads, symptoms of infection, the risk of death, and the situation, such as whether the current response is accurate and effective and how the situation will change. COVID-19 death–related discussion, including death counting, death number, and death rate, dominated this theme.

### Theme 2: Policy and Government Actions

The second most tweeted theme was policy and government actions (92,633/290,764, 31.86%). This theme consisted of six topics: commenting on COVID-19 data reporting being routed away from the CDC (19,098/290,764, 6.57%), the Trump administration's health care policies (18,888/290,764, 6.50%), the policy of reopening schools (18,201/290,764, 6.26%), the CDC not leading the control over COVID-19 (15,010/290,764, 5.16%), the practice of sending COVID-19 patients to nursing homes (10,906/290,764, 3.75%), and the tension between the White House and the CDC (10,530/290,764, 3.62%). Tweets under this theme featured comments that challenged the government’s actions and policies. Many tweets mentioned that the dismissal of the pandemic response team in 2018 and cutting the CDC’s funding weakened the CDC during the COVID-19 pandemic. When the government announced on July 15, 2020, that COVID-19 hospital data would not be reported to the CDC, the number of tweets related to the topic of the CDC not leading the control over COVID-19 for a single day and a single week both set the record (5954 tweets on July 15, 2020, and 13,392 tweets in the week starting on July 15, 2020; see Figure 2 for reference). The dominant voices were complaints against this policy change.

### Theme 3: Response Guidelines

The third most tweeted theme was response guidelines (39,966/290,764, 13.75%), which was about how to respond to COVID-19. This theme covered two topics: the CDC's COVID-19 guidelines with a focus on social distancing and stay-at-home orders (20,032/290,764, 6.89%) and the CDC's recommendation of wearing face masks (19,934/290,764, 6.86%). Both of these topics were highly discussed on Twitter, ranking third and fourth, respectively, according to the number of tweets for a single topic. Most of the tweets under this theme suggested that the CDC's guidelines for individuals, businesses, and other organizations should be followed. Many tweets provided the CDC links to the public for further details; one of the most common CDC links mentioned by Twitter users was the video tutorial released by the CDC about making cloth masks.

### Theme 4: General Opinion About Credibility

The topic of general opinion about credibility of the CDC and other authorities in charge, such as Dr Fauci, Dr Birx, President Donald Trump, the Food and Drug Administration, and the WHO, stands alone as a category of themes, being the fourth most tweeted theme (26,026/290,764, 8.95%) and the second most tweeted topic, trailing only behind the topic of COVID-19 death counting. Different from the other topics, the tweets under this topic did not point to one or a few specific things; instead they usually expressed general opinions and sometimes together with emotions. Words reflecting “credibility,” such as “lie,” “trust,” “listen,” “hoax,” “conspiracy,” “stupidity,” and “fail,” were frequently used by Twitter users. However, it was noted that the negative words did not always point to the CDC; instead, there were a substantial number of tweets grouped under this same theme asking people to stand with the CDC and listen to the scientists (see the representative tweets of this topic in Table 1).

## Discussion

### Principal Findings

Revealed by the quantity of tweets, the public's most prominent concern was death, with over 22.79% of tweets relating to death-related discussion. Previous infoveillance studies of Twitter data in the early period of COVID-19 found that 4.34% of tweets were about death reporting [45] and 10.54% of tweets pertained to deaths caused by COVID-19 [29]. The substantial increase in the death-related discussion with the progression of the pandemic highlighted the urgency of communicating adjusting information to the public, which refers to the information helping them to cope psychologically in threatening situations [46]. Fear and stress were common emotions during the COVID-19 pandemic [29]. Much fear derives from uncertainty and the unknown. Furthermore, the perceived threat in challenging situations motivated people to actively seek information to ease the uncertainty caused by the crisis [47,48]. This explained why a considerable amount of discussion focused on understanding the COVID-19 virus and how the virus has been coped with. In order to put the impact of COVID-19 into perspective, many tweets compared the death rate of COVID-19 with influenza, H1N1 swine flu, Ebola, and pneumonia, which are more familiar to the public. Discussion about the accuracy of COVID-19 death counting and antibody tests also shows the public’s concerns about the current actions of the agencies in charge in response to COVID-19. These findings indicate that in large-scale public health crises such as the COVID-19 pandemic, an imperative component of communication to the public should be informing them of the knowledge of the virus and the factual information about the situations to alleviate fears and confusion. More direct interaction with the public on social media, such as holding an online chat as the CDC had done in the Ebola [20] and Zika outbreaks [13], may also help provide the public with reassurance. In addition, comprehensibility is an important consideration for COVID-19 communication to the public: using language that fits the level of public knowledge helps address the possible misunderstanding of information and avoid the dissemination of misinformation and even rumors.

The majority of the public discussion involved how to act during the COVID-19 pandemic. This echoed past crisis research: in risky environments, the first information that should be conveyed to the public is the information that instructs the public on how to protect themselves in the threatened environments [46]. It also showed that taking actions to prevent the virus from spreading, such as wearing masks and observance of social distancing orders, is a constant topic of the public from the prepandemic period to the peripandemic period [29,45]. The CDC’s instructions on how to act in the context of the COVID-19 pandemic, such as guidelines for reopening, recommendations on wearing masks, and how to make masks, have successfully attracted the public attention as soon as they were released. The public not only spread the guidelines widely on Twitter, but they also tweeted explicitly to urge people to follow the CDC's guidelines by providing official CDC links in their tweets. This reflected the public's urgent need for such information to guide their actions, and they took the instructing information from the CDC very seriously. During the unprecedented crisis of COVID-19, scientific understanding of the virus takes time and keeps evolving. Our study suggested that in the next round of COVID-19 communication, the CDC should continue to strive to translate scientific findings into practical instructions, to provide guidance on how to act for both individuals and organizations, and, finally, to protect the public during the pandemic.

As to the CDC's performance in the COVID-19 response, the public expressed mixed comments. One factor contributing to this may be that the CDC has not played a central role in controlling the pandemic; this deviated from what the CDC had done historically during epidemics [49,50]. Even so, it is noted that the discussions on Twitter showed that most of the Twitter users still looked up to the CDC as the authority in disease control and had great expectations for the CDC to lead the fight against COVID-19. While there were negative wordings (eg, “liars,” “hoax,” “stupidity,” and “failed”) in the public's general opinions about agencies in charge during the pandemic, including the CDC, it was noticeable that many tweets attributed the current performance of the CDC to the government's policy, criticizing the Trump administration's policies and actions for undermining the functioning of the CDC in response to COVID-19. An early study on the outreach efforts of public health authorities on Facebook found that the spike in public response happens in conjunction with specific events [6]. In our study, the trigger event for a record number of tweets was the announcement that the reporting of COVID-19 hospital data would be sent to the Trump administration rather than the CDC, and the dissenting voice dominated the discussion on this topic. To a large extent, this finding is consistent with the findings of a survey study that showed that Americans’ average trust rating for the CDC was significantly higher than that for President Trump [40]. The significance of positive public perception of public health agencies has been receiving increasing recognition [9,10,51,52]. It has been found that greater trust in the CDC was associated with increased knowledge and a lower acceptance of misinformation [40]. The widespread dissemination of the CDC guidelines, as well as the fast speed at which they were circulated, on the one hand reflected the public's urgent need for information as discussed above and, on the other hand, it reflected their trust in the CDC. Even though the CDC's coping so far has not been satisfactory as shown in the tweets, the public's general trust in the CDC is an intangible asset that the CDC can tap into in the next round of the fight against COVID-19.

### Limitations

There are a few limitations to this study. First of all, tweets from accounts marked as private might be missed in the data collection, and tweets generated by bots or fake accounts might not have been filtered. Second, this study identified topics from the public discussion about the CDC but did not examine the temporal variance of topics. Although this is not in our research scope, it may deepen our understanding of how the public changed their focus as time and specific situations during that time changed. Therefore, we highly suggest that future studies put emphasis on the temporal dimension of online public discussion about COVID-19 to get more insight into the formation and variation of the discussion topics. Third, this study did not investigate the public’s emotions shown in the tweets, which is an important dimension of the public discussion. Future research in this line of study may shed light on the public's affective response to the CDC's actions and may inform the CDC about the public's emotions to be addressed during the pandemic. Lastly, Twitter users do not represent the US population [20]. Therefore, as with all social media analyses, findings of this study cannot be generalized to the whole American public.

### Conclusions

In public health crises, social media platforms, such as Twitter, can provide valuable databases for public health agencies to understand the public's concerns, focus of attention, and expectations. The ability of text mining to derive high-quality information from massive data sets is ideal for performing surveillance work. Especially in a protracted pandemic such as COVID-19, quickly and efficiently identifying the topics within the public discussion on Twitter would provide insight for the next round of public health communication in order to mitigate public concerns and avoid the spread of misinformation.

## Abbreviations

ASCII: American Standard Code for Information Interchange
CDC: Centers for Disease Control and Prevention
GUI: graphical user interface
JSON: JavaScript Object Notation
LDA: latent Dirichlet allocation
WHO: World Health Organization
